# Supplementary material for: Climate change impacts on dengue transmission areas in Espírito Santo state, Brazil
Source: Oxf Open Immunol. 2024 Sep 6;5(1):iqae011. doi: 10.1093/oxfimm/iqae011 (PMC11398874; doi:10.1093/oxfimm/iqae011)
Supplement: iqae011_Supplementary_Data [file iqae011_supplementary_data.zip › Manuscript_Oxford_Immunology_Rosa_2024_without_track_changes.docx]

**Title: Climate change impacts on dengue transmission areas in Espírito Santo state, Brazil**

**Running head: Climate change and dengue in Espírito Santo**

Yasmim Barcellos Madeira Rosa^*1^

Henrique Tamanini Silva Moschen^*1,2^

Ana Carolina Loss^3^

Theresa Cristina Cardoso da Silva^4,5^

Ana Paula Brioschi dos Santos^4,5^

Bruna Caetano Pimenta^1^

Julia Sthefany Nunes Zordan^1^

Crispim Cerutti Junior^6,7^

Angelica Espinosa Barbosa Miranda^4,6,7^

Iuri Drummond Louro^8,9^

Débora Dummer Meira^8,9^

Creuza Rachel Vicente^6,7^

*These authors contributed equally and shared the first authorship.

1. School of Biology, Federal University of Espírito Santo, Vitória, Espírito Santo, Brazil

2. Graduate Program in Molecular Biology, University of Brasília, Brasília, Federal District, Brazil

3. Graduate Program in Biological Sciences, Federal University of Espírito Santo, Vitória, Espírito Santo, Brazil

4. Graduate Program in Collective Health, Federal University of Espírito Santo, Vitória, Espírito Santo, Brazil

5. Surveillance Sector, State Health Department of Espírito Santo, Vitória, Espírito Santo, Brazil

6. Graduate Program in Infectious Diseases, Federal University of Espírito Santo, Vitória, Espírito Santo, Brazil

7. Department of Social Medicine, Federal University of Espírito Santo, Vitória, Espírito Santo, Brazil

8. Graduate Program in Biotechnology, Federal University of Espírito Santo, Vitória, Espírito Santo, Brazil

9. Department of Biology, Federal University of Espírito Santo, Vitória, Espírito Santo, Brazil

**Corresponding author:**

Creuza Rachel Vicente

E-mail: vicentecrachel@gmail.com

Address: Department of Social Medicine, Health Science Center, Federal University of Espírito Santo, Marechal Campos Avenue, 1468, Bonfim, Vitória, Espírito Santo, Brazil, Zip-Code: 29047-105

**Abstract**

**Background:** Espírito Santo state, in Brazil, is a dengue-endemic region predicted to suffer from an increase in temperature and drought due to climate change, which could affect the areas with active dengue virus transmission. **Objectives:** Modeling climatic factors and climate change effects in zones suitable for dengue virus transmission in Espírito Santo state, Brazil. **Methods:** Data on dengue reports from 2022 were used to determine climatic variables related to spatial distribution. The climate change projections were generated for the 2030s, 2050s, 2070s, and 2090s for three distinct Shared Socioeconomic Pathways: SSP1-2.6, SSP2-4.5 and SSP5-8.5. A maximum entropy algorithm was used to construct the three models and projections, and the results were used to calculate the ensemble mean. **Results:** Isothermality, the maximum temperature of the warmest month, precipitation of the wettest month, precipitation of the warmest quarter, and annual precipitation impacted the model. Projections indicated a change in areas suitable for dengue virus transmission, varying from -30.44% in the 2070s (SSP1-2.6) to +13.07% in the 2070s (SSP5-8.5) compared to 2022. The coastal regions were consistently suitable in all scenarios. **Conclusion:** Urbanized and highly populated areas were predicted to persist with active dengue transmission in Espírito Santo state, posing challenges for public health response.

**Keywords:** Dengue, Aedes, Arbovirus, Climate effects, Climate change, Global warming.

**Introduction**

In Brazil, the mosquito *Aedes aegypti* is the primary vector of dengue [1]. This disease occurs in seasonal cycles, with higher incidence during warmer and humid seasons [2]. High temperatures accelerate the vector's development and increase its bitting activity, while water accumulation produces breeding sites for its reproduction [3]. Therefore, climate change may contribute to the expansion of dengue transmission areas, even in sub-tropical regions, and could interfere with seasonability, creating more extended periods with favorable conditions for vector reproduction and abundance [4-6].

The epidemiological dynamic of dengue occurrence is also influenced by the human population's herd immunity, with increasing incidence related to the introduction or reintroduction of the four dengue virus serotypes (DENV-1, DENV-2, DENV-3, DENV-4) and their respective genotypes in the population. The cocirculation of different DENVs raises the risk of successive infections, with an immune response that may neutralize or enhance the infection [7]. The interruption of dengue epidemics in Brazil is highly influenced by the development of herd immunity, reducing the transmission rate [8].

In addition to favorable weather and population immune status, unplanned urbanization facilitates the establishment of mosquito breeding sites in Brazil, contributing to the presence of *A. aegypti* in regions with high human population density [8-12]. Nevertheless, even small Brazilian cities have registered dengue epidemics due to their population's lower herd immunity, increasing urbanization, and human mobility, characterizing the territorial expansion of the disease as a trend [13]. Poverty has also been associated with dengue occurrence in this country [14].

Espírito Santo state, in Brazil, had the first report of dengue in 1995 and is an endemic area that suffers from periodic epidemics [15]. All DENV serotypes were already identified in its population, and all the municipalities are at risk of dengue outbreaks since *A. aegypti* is present in them [16]. In addition, an outbreak related to *Aedes albopictus* was registered in a rural area in the north of the state in 2019 [17].

Considering the changes in the climate proposed for the following decades, projections showed an alarming increase in Espirito Santo's temperature, with some areas reaching close to 6 °C in the long term of a more pessimistic scenario [18]. The maximum and minimum temperatures are also very likely to rise in the future, with the maximum going over 3 °C in different parts of the state and the minimum surpassing a similar value by the end of the century [18]. The projections presented a significant rise even in regions with a lower temperature range [18]. Regarding precipitation, the prospect is a drastic reduction throughout the entire state, either in a scenario closer to the present conditions or in a pessimistic scenario. In addition, an increasing frequency of heating days and periods of drought is predicted [18].

Models relating to climatic factors and disease transmission have been employed for infectious diseases, including dengue. Still, more effort is needed to involve low- and middle-income tropical regions in these studies to understand climate change's impact on health [19]. The evaluation of climate change's impact in Espírito Santo state is recent, and no study has analyzed how it will affect dengue transmission locally [18]. The objective of the present study was to evaluate climatic factors associated with regions with active DENV transmission in Espírito Santo state and the influence of climate change on transmission areas in future scenarios.

**Methods**

**Study place:** Espírito Santo state is in the litoral area of the Brazilian southeast region. The state has 78 cities, an area of 46,074.448 km^2^, a population of 3,833,712 inhabitants, and a population density of 83.21 inhabitants/km² in 2022 [20]. The climate is tropical humid, with an average annual temperature of 23 ºC and precipitation exceeding 1,400 millimeters [21]. However, these characteristics vary in nine natural zones with distinct temperatures, relieves, and humidity [22] (Figure 1).

**Data source:** The geolocations used to construct the models were obtained from the reports on dengue in residents of Espírito Santo state with initial symptoms between 1 January 2022 and 31 December 2022 and accessed from the *eSUS Vigilância em Saúde* (eSUS VE). This year was the most recent without dengue epidemic, and the data corresponded to the 12 months of 2022. The precision of the geolocations was checked through Google Maps, inconsistent data were removed, and duplicates were excluded to reduce spatial autocorrelation. The bioclimatic variables were collected from the Worldclim (v2.1) database in a 30-second (~1km) spatial resolution [23].

**Variables and scenarios:** A Variance Inflation Factor (VIF) analysis was performed through the package "usdm" in the software R v4.3 to reduce the correlation between the 19 available variables and select only the ones related to the study area [24, 25]. The future projections were generated based on four time periods in which the bioclimatic variables were available, in a 20-year interval, here represented by 2030s (mean for 2022-2040), 2050s (mean for 2041-2060), 2070s (mean for 2061-2080) and 2090s (mean for 2081-2100). Three distinct Shared Socioeconomic Pathways (SSP) were used for the models. SSP1-2.6 has a more positive outcome for mitigation and adaptation, SSP2-4.5 is the closest to the present situation involving those aspects, and SSP5-8.5 has more negative and drastic expectations toward them [26]. The Hadley General Circulation Model (HadGEM3-GC31-LL), the Earth System Model from the Australian Community Climate and Earth System Simulator (ACCESS-ESM1-5), and the Centre National de Recherches Météorologiques Climate Model (CNRM-CM6-1) were selected for all future climates [27, 28].

The bioclimatic variables selected through the VIF analysis were defined, according to O’Donnell and Ignazio (2012) [29], as follows: Annual Precipitation, measured in millimeters, is a sum of all the values of monthly precipitation throughout the year, and the Maximum Temperature of the Warmest Month, measured in degrees Celsius, displays the maximum temperature of the hottest month over a year or an averaged group of years. For the Precipitation of Wettest Month and Precipitation of Warmest Quarter, both represented in millimeters, the first “identifies the total precipitation that prevails during the wettest month.” while the second “approximates total precipitation that prevails during the warmest quarter.” Lastly, Isothermality, expressed in percentage, quantifies the oscillation of the day-night temperatures relative to the variation of summer-winter for the year [29].

**Spatial analysis:** The maximum entropy algorithm was applied through the "maxnet" function in the "maxnet" package in R to construct the models and projections [25, 30]. This approach uses presence-only geographic data and evaluates how they relate to the areas' climatic profiles to replicate it to generate models and projections. For cross-validation, 10,000 random points of pseudo-absence were generated and used with the presence points in five independent repetitions. The points were divided into 80% for training and 20% for testing. The metrics were the Area Under the Curve (AUC) and the Continuous Boyce Index (CBI) to evaluate the model's performance and accuracy. The values considered were the mean of the five repetitions, and only the models with AUC ≥ 0.7 and CBI > 0.7 were chosen for this study [31, 32]. The threshold chosen to cut the model and projections was the 10^th^ percentile [33]. The results of HadGEM3-GC31-LL, ACCESS-ESM1-5, and CNRM-CM6-1 were used to calculate the ensemble mean.

**Ethics statement:** The study protocol has the approval of the Research Ethics Committee at the Health Science Center of the Federal University of Espírito Santo (opinion number 6.241.070).

**Results**

After removing all the duplicates and inconsistencies, 1,668 geolocated dengue points were found in 2022 and used for modeling. Different climatic variables were suitable for the area considering these points: Isothermality (BIO3), Maximum Temperature of the Warmest Month (BIO5), Precipitation of the Wettest Month (BIO13), Precipitation of the Warmest Quarter (BIO18) and Annual Precipitation (BIO12) (Table 1).

Substantial changes were observed in the expected climate-appropriate zone, comparing the present models (2022) with the projections for the next 80 years. The SSP1-2.6 scenario presented reductions in the climatically suitable area at all intervals, with the largest area loss in the 2070s. The other two scenarios also had mostly decreased values, with none surpassing 20%. For the first 20 years, the loss observed for SSP2-4.5 exceeded 18% compared to the mean of the current models. SSP5-8.5 had a particularly different trend compared to the previous ones, with low values in the first time interval, yet they started to rise in the 2050s and reached a peak of 13% in the 2070s. The optimistic scenario (SSP1-2.6) for dengue reported a decline in the suitable areas and a more intense presence of non-suitable ones in the northwest region of the state for the 2070s. In the same scenario, by the end of the century, the central part of zones climatically suitable were expected to be on the state’s coast. For SSP2-4.5, until 2080, the most significant portion of non-climatically suitable areas remained in the south. Despite that, for the last 20 years, the few cities projected as unsuitable were located mainly in the state’s northwest region. A similar distribution was observed in most of the projections for SSP5-8.5. However, most of the state was expected to be climatically suitable, especially in the HadGEM3-GC31-LL projections (Table 2, Figure 2, Supplementary Table 1).

**Discussion**

The study presented the influence of temperature and precipitation in areas with active DENV transmission in Espírito Santo state in 2022, especially considering the summer season, characterized as the warmest and wettest period, but also the average rain and isothermally along the year. The results also corroborate other studies conducted in different Brazilian states that found an association between higher incidence with increased precipitation frequency and higher average temperature [8, 14, 34-39]. Besides, an investigation found that these parameters had more influence when considering the year's second to fourth months, coinciding with the warmest and wettest period [40]. A systematic review showed an association between the distribution and frequency of dengue cases with precipitation between 83 and 15 millimeters and mean temperature varying from 21 °C to 29.8 °C [41]. In a Brazilian study, an increase of 1 °C in the minimum temperature and 10 millimeters of precipitation was sufficient to affect dengue incidence in the short term [42]. Therefore, even slight changes in these parameters due to climate change could impact the transmission [2].

Climate change may affect Espírito Santo state's areas differently, presenting distinct epidemiological effects in DENV transmission. In the most pessimistic and very likely climate scenario (SSP5-8.5), the regions with active transmission were expected to expand after the 2050s compared to 2022, reaching a peak in the 2070s. Higher temperatures over the year and extreme heat contribute to increased dengue propagation in tropical areas [43]. In Brazil, a previous study demonstrated a similar association, reporting an optimum peak temperature of 31 °C for transmission [44]. In addition, the increasing number of months with temperatures adequate for dissemination or extended periods with temperature abnormalities contributes to dengue outbreaks [45, 46]. Similarly, average and daily range temperatures influence the mosquito vectorial capacity, increasing the potential for a dengue epidemic [47]. High temperatures can also influence *A. aegypti* vectorial competency and survival, as well as the DENV extrinsic incubation period [41, 48]. DENV has the highest thermal optima among mosquito-borne viruses, consisting of 29 °C (95% CI = 28.4 °C - 29.8 °C), with upper thermal limits of 34.5 °C (95% CI = 34.1 °C - 35.8 °C), which contribute to dengue geographical expansion and its increasing seasonal ranges due to climate warming [49].

The areas with lower expansion or higher loss of area adequate for DENV transmission in all scenarios in Espírito Santo state were those located in the mountains in the southwest and south, especially in regions currently considered "cold" (altitudes from 800 to 1,200 meters, minimum average temperature from 7.3 °C to 9.4 °C, and maximum average temperature from 25.3 °C to 27.8 °C) and "mild" (altitudes from 450 to 850 meters, minimum average temperature from 9.4 °C to 11.8 °C, and maximum average temperature from 27.8 °C to 30.7 °C) [22]. Previously, high altitudes acted as a barrier to dengue transmission in Brazil. Nevertheless, these regions have started presenting dengue cases recently, including high incidence rates [46]. In addition, areas in the northwest and north of Espírito Santo state that nowadays experience more than six months of dryness were predicted to have lower expansion or higher area loss in the regions of DENV transmission despite their current maximum temperatures varying from 30.7 °C to 34.0 °C [22]. Mato Grosso state, in Brazil, also showed lower incidence in drier and warmer areas, possibly due to reduced breeding sites [50]. The juvenile phases of the *Aedes* species' lifecycle happen in water, with drought affecting the viability of mosquito development [41]. Nevertheless, using containers for water storage during dry periods may increase breeding sites, with container capacity, utility, and location interfering in the oviposition, being necessary actions to improve knowledge, attitudes, and practices for dengue prevention and control even in dry periods [51, 52]. In Brazil, extreme drought may intensify dengue risk in highly urbanized areas due to intermittent water supply, with the necessity of storing water [53]. Therefore, a previous study found a higher correlation between dengue and annual precipitation in Brazilian areas with improved sanitation [54].

In the 2090s, the increase in dengue transmission areas was lower than in the 2070s in the very likely scenario (SSP5-8.5), mainly in the northwest and north of the state. This already warm and dry area was predicted to experience an increase in temperature of 3.0 °C to 4 °C (SSP2-4.5) or 5.0 °C to 6.0 °C by 2080 (SSP5-8.5). In addition, the reduction of annual rain in 2080 was expected to be 200 to 700 millimeters (SSP2-4.5) or 300 to 1,000 millimeters (SSP5-8.5) in the region [18]. Therefore, the lower availability of natural breeding sites and high temperatures sub-optimal for *A. aegypti* survival and behavior could play a role in the lower expansion after the 2070s [55]. Particularly, northwest and north of Espírito Santo state are suffering a process of desertification, which would pose extreme humidity conditions for *A. aegypti* viability, with loss of body fluid through the spiracles of the respiratory system leading to the death of the mosquito under 60% humidity [56]. However, the adaptative evolution of *A. aegypti* may lead to thermal adaptation in a global warming scenario [57].

The unlikely scenario (SSP1-2.6) predicted reductions in dengue transmission areas over time, but there was a lower reduction in the 2090s compared to the 2070s. This scenario considers decreased carbon dioxide and other greenhouse gas emissions (SSP1-2.6), with temperatures rising lower than 2 °C up to the 2090s [26]. The extremely likely scenario (SSP2-4.5) also predicted consistent area reductions over time between the 2030s and 2090s. Again, in unlikely (SSP1-2.6) and extremely likely scenarios (SSP2-4.5), northwest, north, southwest, and south would concentrate the areas with transmission reduction. Interestingly, the coast will persist as an area with active transmission in all scenarios.

The coast concentrates most of the 730.73 km² of urban areas of the Espírito Santo state, including the Metropolitan Region of Great Vitória and regional centers, such as Linhares in the north and Cachoeiro de Itapemirim in the south [20]. Therefore, beyond the climatic fitness, with high temperatures on the entire coast, urbanization, and high population density play a role in the suitability of these areas for DENV transmission [8]. Therefore, preventive measures for controlling dengue in these areas will continue to be mandatory in all future scenarios.

The study presents limitations since it focused only on analyzing the climatic aspects of areas prone to DENV transmission [58, 59]. However, climate effects on dengue depend on context [60]. Other attributes could influence the disease distribution, such as territory occupation, natural barriers (e.g., topography and vegetation coverage), and microclimate [41]. Also, it was not possible to include meteorological variables in the analysis of future scenarios. In addition, the herd immunity of the local population, the introduction and reintroduction of different DENV serotypes and genotypes, the social determinants of health, and preventive measures could influence the occurrence of dengue in Espírito Santo state. Besides, secondary data on dengue reports were used to evaluate places with active transmission in 2022, which is prone to underreporting. Nevertheless, the results demonstrated how climatic variables and climate change would impact dengue transmission areas in the state, improving preparation for responding to this public health issue.

**Conclusion**

Most climatic variables influencing dengue occurrence in Espírito Santo state were related to the warmest and wettest months. Still, average isothermally and rainfall throughout the year also impacted the models. Due to alterations in temperature and precipitation over 80 years, areas suitable for DENV transmission may comprehend most of the territory of Espírito Santo state, with persistence in the urbanized and densely populated coastal region for all climate change scenarios. Therefore, mitigation and preventive measures for dengue should also consider its future recrudescence, with the necessity of intersectoral collaboration to deal with this issue.

**Conflict of interest statement**

The authors declare no conflict of interest.

**Funding**

None.

**Data availability**

The data underlying this article will be shared on reasonable request to the corresponding author.

**Authors' Contributions**

YBMR (Conceptualization, Methodology, Formal Analysis, Writing – original draft); HT (Conceptualization, Methodology, Formal Analysis, Writing – original draft); ACL (Conceptualization, Methodology, Validation, Writing – original draft); TCCS (Data curation, Writing – review & editing); APBS (Data curation, Writing – review & editing); BCP (Writing – review & editing); JSNZ (Writing – review & editing); CCJ (Writing – review & editing); AEBM (Writing – review & editing); IDL (Writing – review & editing); DDM (Writing – review & editing); CRV (Conceptualization, Methodology, Supervision, Project administration, Resources, Writing – original draft).

**References**

[1] Rodrigues NB, Godoy RSM, Orfano AS *et al*. Brazilian *Aedes aegypti* as a competent vector for multiple complex arboviral coinfections. *J Infect Dis* 2021;**224**:101-108

[2] Campbell KM, Haldeman K, Lehnig C *et al*. Weather regulates location, timing, and intensity of dengue virus transmission between humans and mosquitoes. *PLoS Negl Trop Dis* 2015;**9**:e0003957

[3] Reinhold JM, Lazzari CR, Lahondère C. Effects of the environmental temperature on *Aedes aegypti* and *Aedes albopictus* mosquitoes: A review. *Insects* 2018;**9**:158

[4] Messina JP, Brady OJ, Golding N *et al*. The current and future global distribution and population at risk of dengue. *Nat Microbiol* 2019;**4**:1508-1515

[5] Kraemer MUG, Reiner Jr RC, Brady OJ *et al*. Past and future spread of the arbovirus vectors *Aedes aegypti* and *Aedes albopictus*. *Nat Microbiol* 2019;**4**:854-863

[6] Iwamura T, Guzman-Holst A, Murray KA. Accelerating invasion potential of disease vector *Aedes aegypti* under climate change. *Nat Commun* 2020;**11**:2130

[7] Ribeiro GS, Hamer GL, Diallo M *et al*. Influence of herd immunity in the cyclical nature of arboviruses. *Curr Opin Virol* 2020;**40**:1-10

[8] Azevedo TS, Lorenz C, Chiaravalloti-Neto F. Spatiotemporal evolution of dengue outbreaks in Brazil. *Trans R Soc Trop Med Hyg* 2020;**114**:593-602

[9] Magalhaes T, Chalegre KDM, Braga C *et al*. The endless challenges of arboviral diseases in Brazil. *Trop Med Infect Dis* 2020;**5**:75

[10] Kolimenakis A, Heinz S, Wilson ML. *et al*. The role of urbanisation in the spread of Aedes mosquitoes and the diseases they transmit-A systematic review. *PLoS Negl Trop Dis* 2021;**15**:e0009631

[11] da Silva Neto SR, de Oliveira TT, Teixiera IV *et al*. Arboviral disease record data - dengue and Chikungunya, Brazil, 2013-2020. *Sci Data* 2022;**9**:198

[12] de Oliveira JG, Netto SA, Francisco EO *et al*. *Aedes aegypti* in Southern Brazil: Spatiotemporal distribution dynamics and association with climate and environmental factors. *Trop Med Infect Dis* 2023;**8**:77

[13] Codeco CT, Oliveira SS, Ferreira DAC *et al*. Fast expansion of dengue in Brazil. *Lancet Reg Health Am* 2022;**12**:100274

[14] Bavia L, Melanda FN, de Arruda TB *et al.* Epidemiological study on dengue in southern Brazil under the perspective of climate and poverty. *Sci Rep 2020;***10**:2127

[15] Governo do Estado do Espírito Santo. *Plano estadual de saúde 2020-2023*. Vitória: Secretaria da Saúde, 2019

[16] Governo do Estado do Espírito Santo. *Plano de contingência estadual das arboviroses dengue, Zika e Chikungunya 2021-2023*. Vitória: Secretaria da Saúde, 2021

[17] Rezende HR, Romano CM, Claro IM *et al*. First report of *Aedes albopictus* infected by dengue and Zika virus in a rural outbreak in Brazil. *PloS One* 2020;**15**:e0229847

[18] Reis Junior NC, Nobre CA, Santos JM *et al*. *Mudanças climáticas: efeitos sobre o Espírito Santo*. Jundiaí: Paco, 2023

[19] Van de Vuurst P, Escobar LE. Climate change and infectious disease: A review of evidence and research trends. *Infect Dis Poverty* 2023;**12**:51

[20] Instituto Brasileiro de Geografia e Estatística. *Cidades e estados - Espírito Santo*. https://www.ibge.gov.br/cidades-e-estados/es.html (5 January 2024, date last accessed)

[21] Governo do Estado do Espírito Santo. *Geografia*. https://www.es.gov.br/geografia#:~:text=O%20clima%20do%20Estado%20do,ano%2C%20especialmente%20concentrada%20no%20ver%C3%A3o (5 January 2024, date last accessed)

[22] Feitoza LR, Castro LLF, Resende M *et al*. *Mapa das unidades naturais do estado do Espírito Santo: Informações básicas*. Vitória: Incaper, 2010

[23] Hijmans RJ, Cameron SE, Parra JL *et al*. Very high resolution interpolated climate surfaces for global land areas. *Int J Climatol* 2005;**25**:1965-1978

[24] Naimi B, Hamm NAS, Groen TA *et al*. Where is positional uncertainty a problem for species distribution modeling? *Ecography* 2014;**37**:191-203

[25] R Core Team. *R: A language and environment for statistical computing.* R Foundation for Statistical Computing. https://www.R-project.org/

[26] Tebaldi C, O’Neill BC. Chapter 2 - Climate scenarios and their relevance and implications for impact studies. In: Sillmann J, Sippel S, Russo S (ed). *Climate extremes and their implications for impact and risk assessment*. Elsevier, 2020,11-29

[27] Ryan SJ, Carlson CJ, Mordecai EA *et al*. Global expansion and redistribution of Aedes-borne virus transmission risk with climate change. *PLoS Negl Trop Dis* 2019;**13**:e0007213

[28] Wang, Y, Zhao S, Wei Y *et al*. Impact of climate change on dengue fever epidemics in South and Southeast Asian settings: A modelling study. *Infectious Disease Modelling* 2023; **8**:645-655.

[29] O’Donnell MS, Ignizio DA. Bioclimatic predictors for supporting ecological applications in the conterminous United States, *Geological Survey* 2012; **691:**1-10.

[30] Phillips SJ, Anderson RP, Dudík M *et al.* Opening the black box: an open-source release of *Maxent. Ecography* 2017;**40**:887-893

[31] Fielding AH, Bell JF. A review of methods for the assessment of prediction errors in conservation presence/absence models. *Environ Conserv* 1997;**24**:38-49

[32] Manzoor SA, Griffiths G, Lukac M. Species distribution model transferability and model grain size - Finer may not always be better. *Sci Rep* 2018;**8**:7168

[33] Liu C, Berry PM, Dawson TP *et al*. Selecting thresholds of occurrence in the prediction of species distributions. *Ecography* 2005;**28**:385-393

[34] Yang H, Boldrini J, Fassoni A *et al.* Fitting the incidence data from the city of Campinas, Brazil, based on dengue transmission modellings considering time-dependent entomological parameters. *PLoS One 2016;***11**:e0152186

[35] Stolerman LM, Maia PD, Kutz JN *et al.* Forecasting dengue fever in Brazil: An assessment of climate conditions. *PLoS One* 2019;**14**:e0220106

[36] Xavier LL, Honório NA, Pessanha JFM *et al.* Analysis of climate factors and dengue incidence in the metropolitan region of Rio de Janeiro, Brazil. *PLoS One* 2021;**16**:e0251403

[37] Costa AC, Gomes TF, Moreira RP *et al.* Influence of hydroclimatic variability on dengue incidence in a tropical dryland area. *Acta Trop* 2022;**235**:106657

[38] Marinho RSS, Duro RLS, Mota MTO *et al.* Environmental changes and the impact on the human infections by dengue, Chikungunya and Zika viruses in northern Brazil, 2010-2019. *Int J Environ Res Public Health* 2022;**19**:12665

[39] Fujita DM, Salvador FS, Nali LHDS *et al*. Dengue and climate changes: Increase of DENV-1 in São Paulo/Brazil - 2023. *Travel Med Infect Dis* 2023;**56**:102668

[40] Viana D, Ignotti E. The ocurrence of dengue and weather changes in Brazil: A systematic review. *Rev Bras Epidemiol* 2013;**16**:240-56

[41] Abdullah NAMH, Dom NC, Salleh SA *et al.* The association between dengue case and climate: A systematic review and meta-analysis. *One Health* 2022;**15**:100452

[42] Gomes A, Nobre A, Cruz O. Temporal analysis of the relationship between dengue and meteorological variables in the city of Rio de Janeiro, Brazil, 2001-2009. *Cad Saude Publica* 2012;**28**:2189-2197

[43] Fan J, Wei W, Bai Z *et al.* A systematic review and meta-analysis of dengue risk with temperature change. *Int J Environ Res Public Health* 2014;**12**:1-15

[44] Van Wyk H, Eisenberg JNS, Brouwer AF. Long-term projections of the impacts of warming temperatures on Zika and dengue risk in four Brazilian cities using a temperature-dependent basic reproduction number. *PLoS Negl Trop Dis* 2023;**17**:e0010839

[45] Lee SA, Economou T, Barcellos C *et al.* Effect of climate change, connectivity, and socioeconomic factors on the expansion of the dengue virus transmission zone in 21st century Brazil: An ecological modelling study. *Lancet Planet Health* 2021;**5**:14e0009773

[46] Barcellos C, Matos V, Lana RM *et al*. Climate change, thermal anomalies, and the recent progression of dengue in Brazil. *Sci Rep* 2024;**14**:5948

[47] Liu-Helmersson J, Stenlund H, Wilder-Smith A *et al*. Vectorial capacity of *Aedes aegypti*: Effects of temperature and implications for global dengue epidemic potential. *PloS One* 2014;**9**:e89783

[48] Delrieu M, Martinet JP, O'Connor O *et al*. Temperature and transmission of Chikungunya, dengue, and Zika viruses: A systematic review of experimental studies on *Aedes aegypti* and *Aedes albopictus*. *Curr Res Parasitol Vector Borne Dis* 2023;**4**:100139

[49] Mordecai EA, Caldwell JM, Grossman MK *et al.* Thermal biology of mosquito-borne disease. *Ecol Lett* 2019;**22**:1690-1708.

[50] Machado NG, Lotufo Neto N, Lotufo JBS *et al.* Spatiotemporal dengue fever incidence associated with climate in a Brazilian tropical region. *Geographies* 2023;**3**:673-686

[51] Ahebwa A, Hii J, Neoh KB *et al. Aedes aegypti* and *Aedes albopictus* (Diptera: Culicidae) ecology, biology, behaviour, and implications on arbovirus transmission in Thailand: Review*. One Health* 2023;**16**:100555

[52] Newman EA, Feng X, Onland JD *et al*. Defining the roles of local precipitation and anthropogenic water sources in driving the abundance of *Aedes aegypti*, an emerging disease vector in urban, arid landscapes. *Sci Rep* 2024;**14**:2058

[53] Lowe R, Lee SA, O'Reilly KM *et al*. Combined effects of hydrometeorological hazards and urbanisation on dengue risk in Brazil: A spatiotemporal modelling study. *Lancet Planet Health* 2021;**5**:e209-e219

[54] Oliveira JB, Murari TB, Nascimento Filho AS *et al*. Paradox between adequate sanitation and rainfall in dengue fever cases. *Sci Total Environ* 2023;**860**:160491

[55] Paul KK, Macadam I, Green D *et al*. Dengue transmission risk in a changing climate: Bangladesh is likely to experience a longer dengue fever season in the future. *Environ Res Lett* 2021;**16**:114003

[56] Monintja TCN, Arsin AA, Amiruddin R *et al.* Analysis of temperature and humidity on dengue hemorrhagic fever in Manado municipality. *Gac Sanit 2021*;**35**:S330-S333

[57] Couper LI, Farner JE, Caldwell JM *et al.* How will mosquitoes adapt to climate warming?. *Elife* 2021;**10**:e69630

[58] Xu Z, Bambrick H, Frentiu FD *et al*. Projecting the future of dengue under climate change scenarios: Progress, uncertainties and research needs. *PLoS Negl Trop Dis* 2020;**14**:e0008118

[59] Bhatia S, Bansal D, Patil S *et al*. A retrospective study of climate change affecting dengue: Evidences, challenges and future directions. *Front Public Health* 2022;**10**:884645

[60] Kirk D, Straus S, Childs ML *et al.* Temperature impacts on dengue incidence are nonlinear and mediated by climatic and socioeconomic factors: A meta-analysis. *PLOS Clim* 2024;**3**:e0000152

**Table 1. Bioclimatic variables related to dengue and their corresponding permutation importance.**

| **Bioclimatic variable** | **Permutation importance** | **SD** |
| --- | --- | --- |
| Annual Precipitation | 5.0 | ±0.002 |
| Maximum Temperature of Warmest Month | 14.7 | ±0.005 |
| Precipitation of Wettest Month | 16.0 | ±0.002 |
| Precipitation of Warmest Quarter | 30.3 | ±0.008 |
| Isothermality | 34.0 | ±0.003 |

Permutation importance: variable's impact on constructing the models. SD: standard deviation. AUC = 0.75. CBI = 0.98.

**Table 2. Ensemble mean of the changes in climatically favorable areas for dengue in 2030s, 2050s, 2070s, and 2090s compared to 2022, in three climate change scenarios**

| **Climate change scenario** | **2030s** | | **2050s** | | **2070s** | | **2090s** | |
| --- | --- | --- | --- | --- | --- | --- | --- | --- |
|  | **Change (%)** | **Area (km^2^)** | **Change (%)** | **Area (km^2^)** | **Change** | **Area (km^2^)** | **Change (%)** | **Area (km^2^)** |
| **SSP1-2.6** | –7.00 | 28,569.80 | –10.07 | 27,621.60 | –30.44 | 21,341.13 | –8.57 | 28,061.07 |
| **SSP2-4.5** | –18.37 | 25,072.93 | –10.78 | 27,404.80 | –7.030 | 28,569.47 | –2.27 | 30,037.53 |
| **SSP5-8.5** | –11.53 | 27,185.93 | +3.65 | 31,847.17 | +13.07 | 34,738.77 | +9.73 | 33,724.83 |

Models used in the ensemble mean calculation: Hadley General Circulation Model (HadGEM3-GC31-LL), Centre National de Recherches Météorologiques Climate Model (CNRM-CM6-1), and Earth System Model from the Australian Community Climate and Earth System Simulator (ACCESS-ESM1-5). The ensemble mean area in 2022 was 30,704.3 km^2^. Climate change scenarios: SSP1-2.6 - unlikely; SSP2-4.5 - extremely likely, SSP5-8.5 - very likely. Change (%): + increase in the area compared to 2022; - decrease in the area compared to 2022.

**Figure 1. Natural zones of Espírito Santo state, Brazil**

Shapefile source: https://geobases.es.gov.br/links-para-mapas

**Figure 2. Suitable areas for dengue transmission in Espírito Santo state in the 2030s, 2050s, 2070s, and 2090s in the climate change scenarios SSP1-2.6, SSP2-4.5, SSP5-8.5 compared to 2022**

Orange (A): Hadley General Circulation Model (HadGEM3-GC31-LL). Green (B): Centre National de Recherches Météorologiques Climate Model (CNRM-CM6-1). Purple (C): Earth System Model from the Australian Community Climate and Earth System Simulator (ACCESS-ESM1-5).
