# Supplementary material for: Climate change impacts on dengue transmission areas in Espírito Santo state, Brazil
Source: Oxf Open Immunol. 2024 Sep 6;5(1):iqae011. doi: 10.1093/oxfimm/iqae011 (PMC11398874; doi:10.1093/oxfimm/iqae011)
Supplement: iqae011_Supplementary_Data [file iqae011_supplementary_data.zip › Supplementary Table 1.docx]

**Supplementary Table 1. Suitable areas for dengue transmission in Espírito Santo state in the 2030s, 2050s, 2070s, and 2090s in the climate change scenarios SSP1-2.6, SSP2-4.5, SSP5-8.5 compared to 2022**

| **Climate change scenario** | **2030s** | | **2050s** | | **2070s** | | **2090s** | |
| --- | --- | --- | --- | --- | --- | --- | --- | --- |
|  | **Change (%)** | **Area (km^2^)** | **Change (%)** | **Area (km^2^)** | **Change (%)** | **Area**  **(km^2^)** | **Change (%)** | **Area (km^2^)** |
| **HadGEM3-GC31-LL** | | | | | | | | |
| **SSP1-2,6** | 23.1 | 38,071.1 | 8.5 | 33,554.3 | -27.6 | 22,375.9 | -0.9 | 30,648.7 |
| **SSP2-4,5** | 1.1 | 31,273.4 | 10.2 | 34,088.5 | 31.0 | 40,501.6 | 29.3 | 40,000.2 |
| **SSP5-8,5** | 9.3 | 33,819.8 | 29.1 | 39,943.2 | 40.4 | 43,425.9 | 38.9 | 42,974.6 |
| **CNRM-CM6-1** | | | | | | | | |
| **SSP1-2,6** | -4.4 | 29,166.8 | 12.4 | 34,306.2 | -0.01 | 30,507.7 | 26.4 | 38,559.8 |
| **SSP2-4,5** | -11.7 | 26,922.1 | -5.6 | 28,815.5 | -3.9 | 29,326.8 | -2.0 | 29,897.9 |
| **SSP5-8,5** | -17.4 | 25,191.6 | 0.4 | 30,647.6 | 12.5 | 34,339.1 | -8.2 | 28,001.1 |
| **ACCESS-ESM1-5** | | | | | | | | |
| **SSP1-2,6** | -39.7 | 18,471.5 | -51.1 | 15,004.3 | -63.7 | 11,139.8 | -51.2 | 14,974.7 |
| **SSP2-4,5** | -44.5 | 17,023.3 | -37.0 | 19,310.4 | -48.2 | 15,880.0 | -34.1 | 20,214.5 |
| **SSP5-8,5** | -26.5 | 22,546.4 | -18.6 | 24,950.7 | -13.7 | 26,451.3 | -1.5 | 30,198.8 |

Total area: Hadley General Circulation Model (HadGEM3-GC31-LL) = 30,926.3 km^2^. Centre National de Recherches Météorologiques Climate Model (CNRM-CM6-1) = 30,511.3 km^2^. Earth System Model from the Australian Community Climate and Earth System Simulator (ACCESS-ESM1-5) = 30,675.2 km^2^.
